# Development of a Carbon Nanotube-Enhanced FAS Bilayer Amphiphobic Coating for Biological Fluids

**DOI:** 10.3390/nano13243138

**Published:** 2023-12-14

**Authors:** Sumona Paul, Lingfen Rao, Louis H. Stein, Arash Salemi, Somenath Mitra

**Affiliations:** 1 Department of Chemistry and Environmental Science, New Jersey Institute of Technology, 161 Warren Street, Newark, NJ 07102, USA; sp2652@njit.edu (S.P.); lingfen.rao@njit.edu (L.R.); 2 Northern Department of Cardiothoracic Surgery, RWJBarnabas Health, 94 Old Short Hills Road, Livingston, NJ 07039, USA; louis.stein@rwjbh.org (L.H.S.); arash.salemi@rwjbh.org (A.S.); 3 Department of Surgery, Rutgers New Jersey Medical School, 185 S Orange Ave, Newark, NJ 07103, USA

**Keywords:** nanomaterial coatings, biomedical devices, hydrophobicity, amphiphobic surface, carbon nanotubes, fluoroalkyl silane

## Abstract

This study reports the development of a novel amphiphobic coating. The coating is a bilayer arrangement, where carbon nanotubes (CNTs) form the underlayer and fluorinated alkyl-silane (FAS) forms the overlayer, resulting in the development of highly amphiphobic coatings suitable for a wide range of substrates. The effectiveness of these coatings is demonstrated through enhanced contact angles for water and artificial blood plasma fluid on glass, stainless steel, and porous PTFE. The coatings were characterized using Fourier-transform infrared spectroscopy (FTIR), scanning electron microscopy (SEM), thermogravimetric analysis (TGA), atomic force microscopy (AFM), and contact angle (CA) measurements. The water contact angles achieved with the bilayer coating were 106 ± 2°, 116 ± 2°, and 141 ± 2° for glass, stainless steel, and PTFE, respectively, confirming the hydrophobic nature of the coating. Additionally, the coating displayed high repellency for blood plasma, exhibiting contact angles of 102 ± 2°, 112 ± 2°, and 134 ± 2° on coated glass, stainless steel, and PTFE surfaces, respectively. The presence of the CNT underlayer improved plasma contact angles by 29%, 21.7%, and 16.5% for the respective surfaces. The presence of the CNT layer improved surface roughness significantly, and the average roughness of the bilayer coating on glass, stainless steel, and PTFE was measured to be 488 nm, 301 nm, and 274 nm, respectively. Mechanistically, the CNT underlayer contributed to the surface roughness, while the FAS layer provided high amphiphobicity. The maximum effect was observed on modified glass, followed by stainless steel and PTFE surfaces. These findings highlight the promising potential of this coating method across diverse applications, particularly in the biomedical industry, where it can help mitigate complications associated with device–fluid interactions.

## 1. Introduction

In the clinical realm, a multitude of medical devices necessitate interaction with blood, plasma, or various other biological fluids. Examples include intravenous cannulas, peripherally inserted central venous catheters (CVCs), coronary stents, prosthetic heart valves, ventricular assist devices (VADs), dialysis circuits, cardiopulmonary bypass (CPB) circuits, and extracorporeal membrane oxygenator (ECMO) circuits [1,2]. The performance of these devices relies heavily on how their surfaces interact with bodily fluids. For example, device-related thrombus poses significant challenges through potentially obstructing the implanted device, causing thromboembolism in critical organs like the brain or lungs. Consequently, the development of robust biofluid-compatible surfaces that effectively prevent or minimize surface interactions remains a critical task [1,3,4].

One of the main factors that restrict the clinical usability of blood/plasma-contacting biomaterials is hemocompatibility. Contact between blood, a complex mixture made up of 55% plasma, 44% erythrocytes, and 1% leukocytes and platelets, and biomaterials can elicit immunogenic and thrombogenic responses [2]. To address this, newly developed materials require extensive analysis to avoid adverse sequelae of blood–bio surface interaction. Therefore, materials with smart design characteristics are urgently needed to limit the adhesion between biological fluids and the surface [2,3,4,5,6].

To address these challenges, various strategies have been employed [1,3,6,7,8,9,10,11,12,13,14,15,16,17,18]. One option has been the introduction of hydrophobic and superhydrophobic surfaces to medical equipment to enhance its compatibility with body fluids. The hierarchical structures present on hydrophobic surfaces effectively reduce adhesion forces, resulting in improved hemocompatibility. On superhydrophobic surfaces, blood/plasma move swiftly through the boundary layer, minimizing the chances of blood cells colliding with the surface. This leads to reduced adhesion and deformation of blood cells [19].

The contact angle, which represents the angle formed between the liquid–vapor interface and the contour of the contact surface, serves as a reliable indicator of hydrophobicity. In general, contact angles ≤ 90° are indicative of hydrophilic surfaces, while angles ≥ 90° suggest hydrophobic properties [20]. Various materials, including glass, polymers, metals, nonmetal oxides, and composites have been used to develop hydrophobic surfaces. These hydrophobic and superhydrophobic surfaces have been engineered for diverse applications such as self-cleaning, anti-fogging, anti-fouling, oil–water separation, anti-corrosion, anti-icing, antibacterial properties, drag reduction, and fundamental scientific research [21,22,23,24,25,26,27]. Biopolymers such as polyurethane (PU), polyvinylidene fluoride (PVDF), polydimethylsiloxane (PDMS), polymethyl methacrylate (PMMA), polyethylene (PE), polypropylene (PP), polylactic acid, and polycaprolactone (PCL) have been explored as potential substrates and coating materials for medical applications [3].

A superhydrophobic surface exhibits an even higher contact angle ≥ 150° [1,28]. As per the Cassie–Baxter model, surface roughness, in addition to the coating itself, can further enhance hydrophobicity [29]. Nanoparticles, including titanium oxides (TiO_2_), zinc oxide (ZnO), and silicon dioxide (SiO_2_), have shown promise as coating materials for hydrophobic surfaces [2,3,10,21,22,28]. For instance, ZnO nanowires have been used via drop coating on materials such as glass, quartz, silicone, and PDMS to create hydrophobic surfaces for biomedical applications. Polysilsesquioxane (PSQ) composites have been utilized to generate superhydrophilic and superhydrophobic monolayer coatings with antibacterial properties through a straightforward one-step dipping method on silicon wafers and glycol-modified polyethylene terephthalate (PETG) sheets [23]. While a hydrophobic surface repels water, it can be contaminated by organic liquids with lower surface tensions. To address the challenge posed by the high wettability of organic liquids on surfaces, there is a need for amphiphobic surfaces that can repel other components as well [30,31].

The cylindrical nanostructure of carbon nanotubes (CNTs) is capable of providing micro/nano roughness, contributing to their unique properties. Additionally, CNTs can undergo functionalization to modify their surface hydrophobicity or hydrophilicity, making them highly adaptable for tailored applications [19,32,33,34,35,36,37,38,39]. Due to their exceptional characteristics, CNTs have garnered significant attention in biological applications, leading to extensive research on various functionalized forms [40,41,42]. The superior viral removal efficiency demonstrated by carbon nanotubes (CNTs) and their functionalized analogs highlights their potential in antiviral applications [41]. Additionally, the application of CNTs in in vivo cancer treatment within a mouse model has demonstrated encouraging potential. CNT-based sensors have been developed to detect biological species, including proteins and DNA [43]. Notably, recent studies have focused on conjugating biological and bioactive species, such as proteins, carbohydrates, and nucleic acids, with carbon nanotubes. These nanotube bioconjugates hold great promise for advancing the utilization of carbon nanotubes in biomedical technology [42,44,45,46,47].

The field of high-performance and highly functional polymeric materials is experiencing increasing interest, with a focus on developing composite coatings that combine organic polymers with different nanoparticles [1,2,4,15,17,26,28,48]. Among the noteworthy materials are fluorinated polymers, well-known for their excellent chemical and thermal stability, low surface energy, as well as optical and dielectric properties [49,50,51,52]. To achieve superhydrophobic surfaces, researchers have explored the use of fluorinated alkyl-silane (FAS) compounds [53,54]. Recent studies have demonstrated the successful development of biocompatible superhydrophobic surfaces by applying FAS coatings on substrates with spin-cast silica films, making them well-suited for biomedical applications [55,56,57]. Additionally, incorporating zinc oxide and copper nanoparticles into FAS coatings has exhibited robust bactericidal properties without harming mammalian cells, while simultaneously preventing the adherence of blood components and bacteria [25,58].

The primary goal of this study was to create and study a bilayer coating by combining carbon nanotubes (CNTs) with fluorinated alkyl-silane (FAS) to achieve an amphiphobic surface capable of effectively repelling biological fluids. The FAS layer was intended to provide hydrophobicity to the coating, while the incorporation of CNTs aimed to enhance the surface roughness. Another key objective was to assess the performance of this novel coating on three different substrates: glass, metal, and poly(tetrafluoroethylene) (PTFE). By evaluating the coating’s behavior on these diverse surfaces, we aim to understand its potential applicability in biomedical settings.

## 2. Experimental

### 2.1. Materials

1H, 1H, 2H, 2H-Perfluorooctyltriethoxysilane, 97% (FAS), and ethanol were supplied by Thermo Fisher Scientific (HANOVER PARK, IL, USA). Poly(vinylidene fluoride-co-hexafluoropropylene) (PVDF-HFP, Mw: 455,000 g/mol) was purchased from Sigma-Aldrich (St. Louis, MO, USA). Acetone and detergent (Alconox, Sigma-Aldrich, St. Louis, MO, USA) were obtained from Sigma Aldrich (St. Louis, MO, USA). Multiwalled carbon nanotubes (CNTs) were purchased from Cheap Tubes Inc., Brattleboro, VT, USA. The average diameter of the CNTs was ~30 nm with a length of 15 μm. Deionized water (Barnstead 5023, Dubuque, IA, USA) and artificial plasma fluid (Biochemazone, Edmonton, AL, Canada) were used in all experiments. Three types of materials were used as coating surfaces, including steel, glass, and PTFE. Clear glass (0.22 mm × 0.22 mm) from Globe Scientific Inc. and stainless-steel sheet (~0.05 mm thickness) from MnsbestDeal2000, Shenzhen, China, were used in this study. Flat polytetrafluoroethylene (PTFE) was used with PP nonwoven fabric (ANNOW, Wuhan, China), which had a pore size of 0.05 µm and 62% porosity.

### 2.2. Preparing of Hydrophobic Coating Suspensions 

The suspension used for preparing the coatings comprised two steps. In the first step (Step 1), a predetermined quantity of CNTs (ranging from 5 to 20 mg) was dispersed in acetone (approximately 5–10 mL). This dispersion was then subjected to sonication in a glass beaker for 2 h to ensure proper dispersion. Next, 1–4 pellets of PVDF HFP (~0.2–0.5 g) were added to the CNT dispersion, and the mixture was sonicated for an additional hour to achieve a homogeneous dispersion. The purpose of this sonication treatment was to disperse CNTs and enhance their even distribution. The specific concentrations of CNTs and PVDF were adjusted based on the requirements of each material being used in the coating preparation process.

To prepare the second part (Step 2) of the coating, a solution of FAS was created in a glass beaker using a magnetic stirrer at a rate of 300 rpm for 20 min to ensure homogeneity. The FAS was added to ethanol while continuously stirring, resulting in a solution (Step 2) with a concentration of 3% (*v*/*v*). This step generated a well-mixed and consistent FAS solution to be used in the subsequent coating process.

### 2.3. Surface Coating

Three substrates, namely stainless steel, glass, and PTFE, were selected. The glass and stainless-steel plates were first pretreated by immersing them in detergent in an ultrasonic bath for 20 min, then rinsed with demineralized water and dried in air at room temperature for 30 min. They were again immersed in acetone and sonicated for another 20 min. Finally, they were rinsed with demineralized water and dried in air at room temperature.

**Figure 1:**
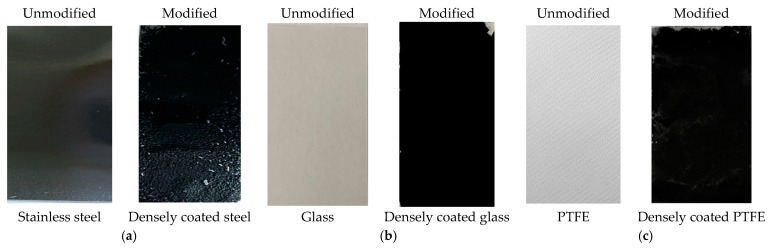
Photographs of the samples before and after modification with FAS–CNT: (**a**) steel, (**b**) glass, and (**c**) PTFE.

The design of the coating consists of a top and bottom layer, as shown in Figure 1.

**Figure 2:**
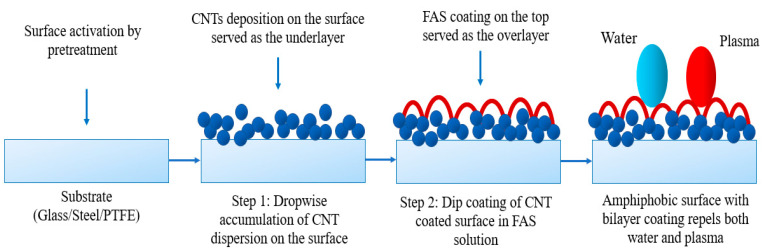
Schematic of bilayer coating arrangement.

The base layer was prepared by the dropwise addition of CNT dispersion onto the steel and glass substrates, while vacuum-assisted coating was applied to the PTFE substrates. The concentration of CNT (~5 mg) and PVDF HFP (~0.085 g) were the same for steel and glass, while the quantity of CNT (20 mg) and PVDF (~0.2 g) was higher for PTFE to ensure a dense non-porous coating. After casting the CNT dispersion onto the materials, the samples were dried in an oven at 80 °C for 2 h. For stainless steel, the CNT dispersion was cast onto pre-treated steel and cured in a preheated oven at 120 °C for 2 h. Once the base coat was ready, these CNT-coated samples were immersed in a 3% (*v*/*v*) FAS solution for 3 h to allow dip coating, and then heated in an oven at 160 °C for 2 h to form the top layer. This cycle was repeated three times to complete the surface coating. After that, the samples were ready for experiments. Figure 1 shows the three different samples of stainless steel, glass, and PTFE before and after modification. The FAS layer was transparent and is not presented here for brevity. The overall bilayer coating arrangement to develop amphiphobic surface is briefly illustrated in Figure 2.

Plasma, a major component of blood, is made up of 6–8% dissolved solids, such as proteins (e.g., serum albumins, globulins, and fibrinogen), glucose, electrolytes, and others. The amphiphobic surface repelled water and plasma fluid simultaneously upon contact. The surface was able to repel plasma proteins, which tend to bind strongly to many surfaces. The amphiphobic nature is expected to inhibit platelet aggregation and cell adhesion, two crucial steps in the process of preventing blood clotting [30,31,59,60,61,62]. Additionally, fluorination of the surface by fluoropolymers decreased its attraction to water and plasma, allowing for the formation of high contact angles on the surface, as evident from the contact angle experiments.

### 2.4. Characterization

The morphology of the surface coatings was characterized using a scanning electron microscope (SEM, model JSM-7900F, JEOL USA Inc., Peabody, MA, USA). The static water contact angle and plasma contact angle were measured to study the change in hydrophobicity of the coated surface. Water droplets (4 μL) were dropped onto the membrane surface using a Hamilton micro-syringe (0–10 μL), and a stage-mounted video camera was used to record pictures of the droplets [63,64,65]. A minimum of five readings were taken, and the average contact angle was calculated. Attenuated total reflection Fourier transform infrared (ATR-FTIR) spectra of the samples were collected via an Agilent Cary 610/620 FTIR instrument in the 400–4000 cm^−1^ region. The thermal stability of the samples was investigated using the Perkin Elmer Pyris 7 TGA instrument at an isothermal heating rate of 10 °C/min in air. The surface topography and roughness of the membranes were determined by Bruker Atomic Force Microscopy Dimension Icon under ambient conditions using the silicon nitride cantilever containing a silicon probe with a frequency of 70 kHz and a tip radius of 2 nm for a scan area of 10 μm × 10 μm.

## 3. Results and Discussion

**Figure 3:**
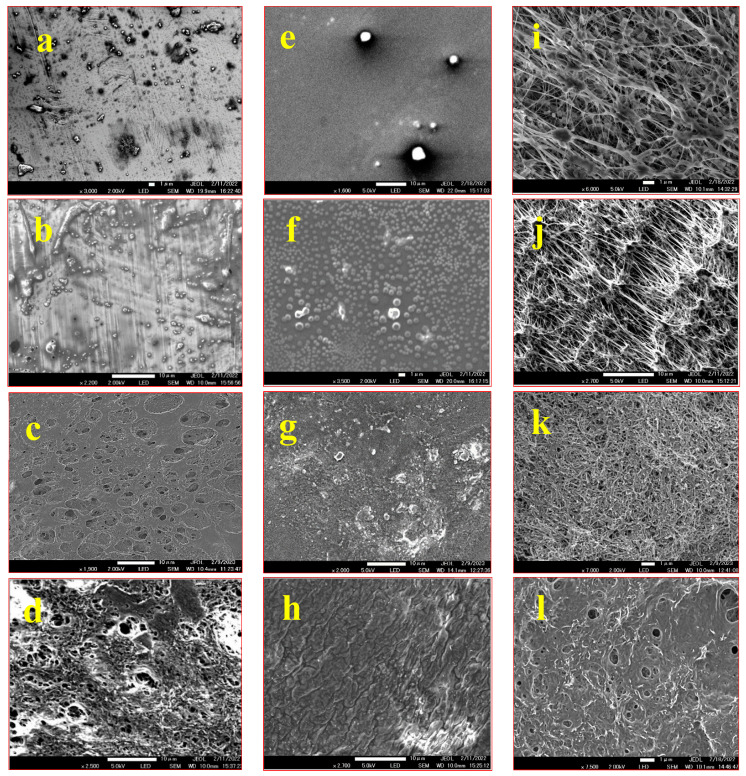
SEM images of steel, glass, and PTFE samples: (**a**) unmodified steel; (**b**) steel with FAS coating; (**c**) steel with CNT coating; (**d**) steel with FAS–CNT coating; (**e**) unmodified glass; (**f**) glass with FAS coating; (**g**) glass with CNT coating; (**h**) glass with FAS–CNT coating; (**i**) unmodified PTFE; (**j**) PTFE with FAS coating; (**k**) PTFE with CNT coating; (**l**) PTFE with FAS–CNT coating.

The morphology of the coated steel, glass, and PTFE substrates was examined using scanning electron microscopy (SEM), and the corresponding images of the substrates coated solely with FAS and the composite FAS–CNT coating are displayed in Figure 3. The surfaces coated with the FAS–CNT composite showed improved topography compared to those coated solely with FAS. The carbon nanotubes (CNTs) were distributed on the surface, creating a hierarchical structure and contributing to micro/nano roughness due to their high aspect ratio structures [32,33,37]. The combined effect of the surface morphology and low surface energy resulted in the coating displaying hydrophobic characteristics. Notably, for PTFE, a uniform distribution of a significant amount of CNTs was observed within the substrate pores, rendering the PTFE non-porous with potentially enhanced hydrophobicity. This composite coating exhibited promising potential for creating superhydrophobic surfaces on different substrates.

**Figure 4:**
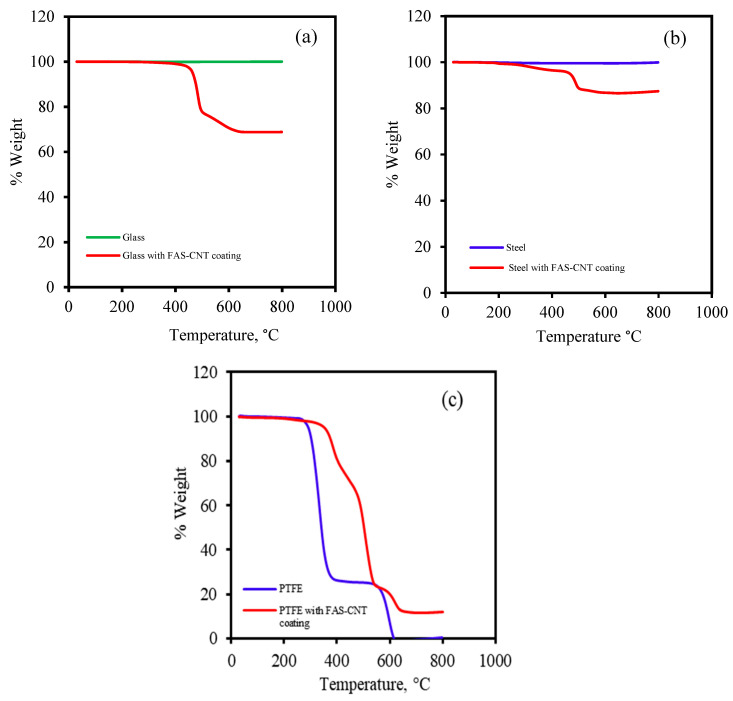
TGA curves for different samples: (**a**) glass (**b**) steel, and (**c**) PTFE.

Thermal gravimetric analysis (TGA) was performed to study the thermal stability of modified and unmodified samples using the Perkin Elmer Pyris 7 TGA system. Figure 4 shows the TGA curves for the modified samples in comparison with the unmodified samples. Figure 4a represents the TGA curve for unmodified glass and FAS–CNT-modified surfaces in comparison. The unmodified glass did not show any degradation in percent weight, while the modified surface exhibited degradation from 400 to 800 °C. The degradation was due to the presence of FAS and CNT on the surface. Similarly, the unmodified steel did not show any change in percent weight, but the modified surface showed changes from 350 to 800 °C due to FAS. Meanwhile, the FAS–CNT-modified PTFE surface showed degradations at different zones of temperatures, which is similar to the unmodified PTFE substrate.

The PTFE substrate was supported by polypropylene; therefore, the unmodified PTFE substrate showed two regions of degradation. The first degradation was for polypropylene, starting from 250 to 350 °C, and the second degradation zone was from 430 to 650 °C. For the modified FAS–CNT-coated surface, there were three degradation zones, namely polypropylene, PTFE, and FAS. In the 250 to 500 °C temperature range, FAS and polypropylene degraded, while PTFE started degrading at 550 °C and continued up to 700 °C. It was observed that the presence of CNTs provided additional thermal stability to the modified samples. The results were in line with what we have reported previously [32,33,63,64,65].

**Figure 5:**
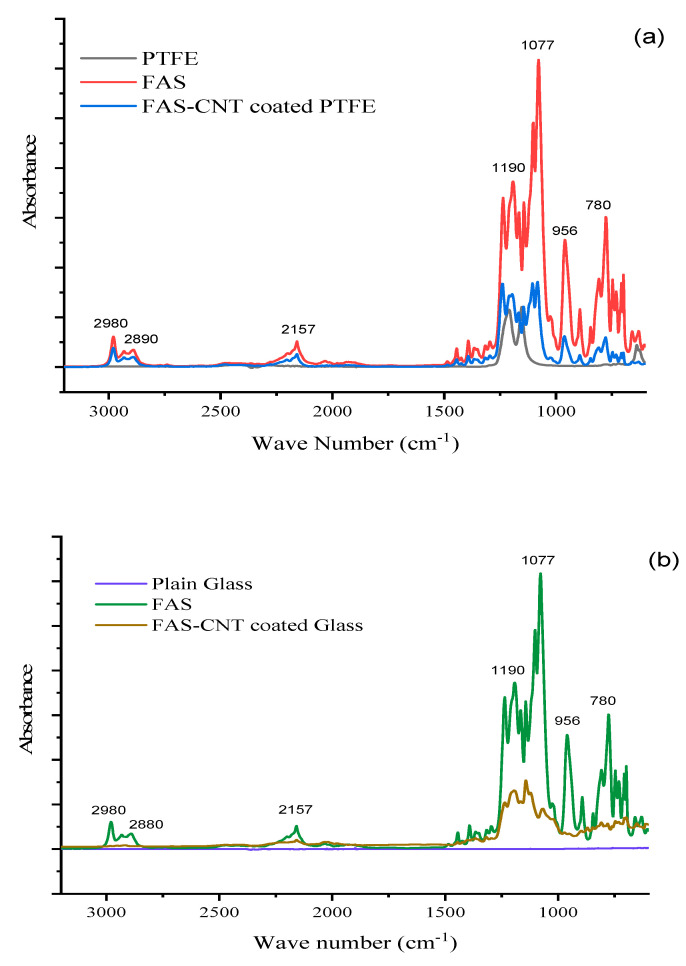

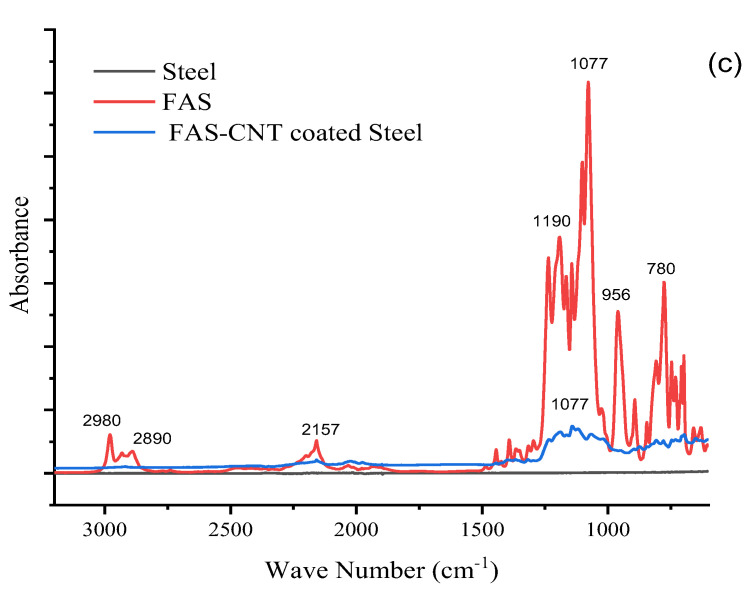
FTIR spectra of different samples; (**a**) PTFE, (**b**) glass and (**c**) steel.

The FTIR spectra of the base PTFE, FAS solution, and modified surfaces are shown in Figure 5a, Figure 5b, and Figure 5c, respectively. Figure 5a shows the spectra of the surface with FAS–CNT coating over the base PTFE, Figure 5b shows the FAS–CNT-coated glass surface, and Figure 5c shows the FAS–CNT-coated metal surface. Glass and metal surfaces do not show any significant peaks in the FTIR spectra. Meanwhile, the base PTFE membrane shows peaks corresponding to C–H and C–F bonds. Some of the characteristic peaks in Figure 5a–c are observed around 780, 956, 1077, 1190, 2890, and 2980 cm^−1^ for FAS and FAS-coated surfaces [66]. Also, a small peak at 2157 cm^−1^ appears in all our samples due to the interference of atmospheric CO_2_ in the instrumentation. The peak at 1077 cm^−1^ is due to the asymmetric stretching vibration of Si–O bonds. The peak at 956 cm^−1^ is attributed to the C–F bond stretching. Another small peak appearing at around 780 cm^−1^ is associated with the bending mode of Si–O bonds. Another two peaks at 2980 and 2890 cm^−1^ symbolize the presence of symmetrical and asymmetrical C–H stretching bonds [13,18,66]. There are indications of the presence of long-chain alkyl groups on the surface. The most significant peak, appearing at 1190 cm^−1^, signifies the presence of a Si–O–C bond, which is a characteristic bond in the FAS structure [66]. This symbolizes the presence of FAS on the surface. The presence of FAS is also confirmed by the TGA analysis.

**Figure 6:**
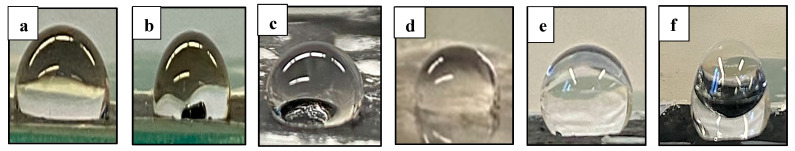
Contact angles on modified surfaces of steel, glass, and PTFE. Contact angle on modified steel surface for: (**a**) water and (**b**) plasma. Contact angle on modified glass surface for: (**c**) water and (**d**) plasma. Contact angle on modified PTFE surface for: (**e**) water and (**f**) plasma.

The wettability of both uncoated and coated samples was examined via contact angle measurements using both water and plasma (Figure 6) [32,33]. Unmodified glass and metal exhibited hydrophilic behavior with a water contact angle of 20 ± 2° and 80 ± 2°, respectively, while the PTFE showed hydrophobicity with a contact angle of 109 ± 2°. Figure 6 shows the images of contact angles for water and plasma on the modified steel, glass, and PTFE.

The results summarized in Table 1 indicate that the presence of CNT and FAS together in the coated samples dramatically increased the contact angle. The contact angle improvement was significantly manifested for both water and plasma, which also contributes to the enhancement in surface hydrophobicity. Note that the change in the contact angles for surfaces modified with FAS and CNT confirms that carbon nanotubes have a better capability of orienting fluoroalkyl groups on the surface and increasing hydrophobic properties.

The surface energy of the coated materials was lowered, and they repelled water and plasma as evidenced by the measured contact angles. The increase in hydrophobicity was highest for the glass, followed by the metal. The microstructures on the glass surface developed roughness, which helped increase the contact angle of water droplets compared to the way water instantly spreads on the uncoated glass and becomes sticky due to the hydrophilic nature of the surface [18]. PTFE was quite hydrophobic to begin with; it repelled both water and plasma. However, the coating improved repellency by 29.4% for water and 31.3% for plasma.

**Figure 7:**
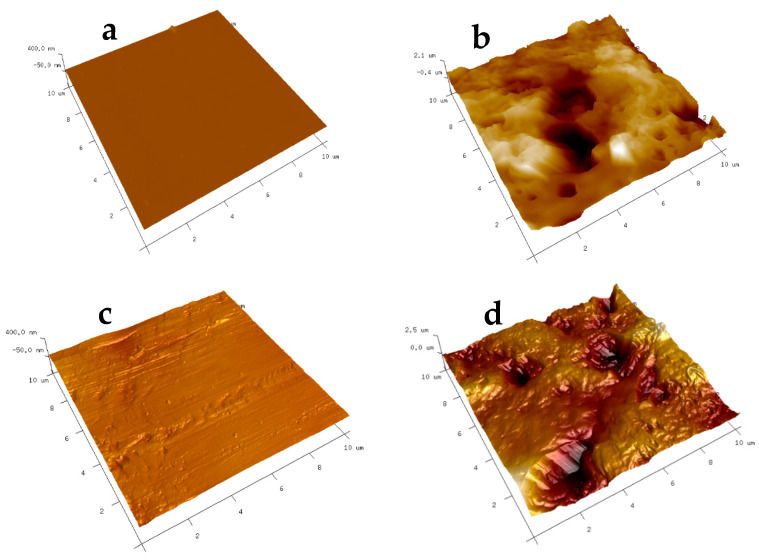

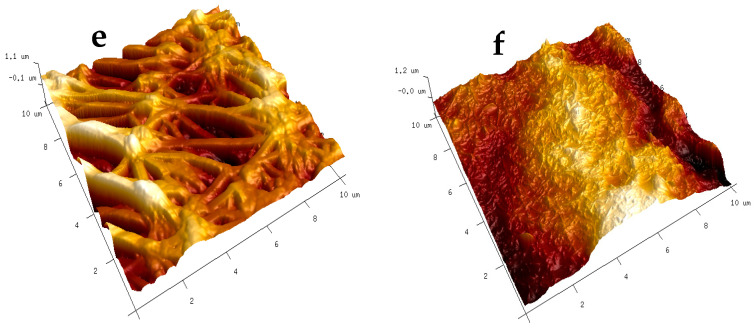
AFM images of modified surfaces: (**a**) glass-coated with FAS; (**b**) glass-coated with FAS–CNT; (**c**) steel-coated with FAS; (**d**) steel-coated with FAS–CNT; (**e**) PTFE-coated with FAS; and (**f**) PTFE-coated with FAS–CNT.

The AFM analysis results, as presented in Figure 7, depict the surface roughness of various substrates with and without CNT coatings. The scanning area for all substrates was 10 μm × 10 μm. Two different locations were scanned for each sample to ensure representative measurements. The average roughness parameters, Ra (average surface roughness), and Rq (root mean square average), were analyzed using the NanoScope Analysis (v1.40r1) Software, and the results are summarized in Table 2.

It is evident from the data that the substrates coated with FAS–CNT exhibit significantly higher surface roughness compared to those with FAS only. This increased roughness is attributed to the incorporation of carbon nanotubes in the CNT underlayer [67,68]. Among the modified surfaces, the presence of CNTs contributes to the highest surface roughness in the modified glass, followed by modified steel and PTFE. These findings demonstrate that the addition of carbon nanotubes in the coating significantly impacts the surface roughness of the substrates, which can have implications for their hydrophobicity and adhesion properties. The higher roughness parameters in Table 2 also correspond to the enhancement in contact angles in Table 1.

## 4. Conclusions

A bilayer amphiphobic coating was successfully created by employing CNT and FAS, using drop coating followed by dip coating. The presence of both FAS and CNT was confirmed using FTIR analysis, while contact angle measurements demonstrated the coating’s strong water and plasma repellency. SEM images revealed the formation of hierarchical structures on the surface after coating with CNT and FAS. Through TGA analysis, it was established that the coated samples exhibited thermal stability up to temperatures of 300–500 °C, proving them suitable for various high-temperature applications. Surface roughness improvement was observed through AFM analysis. The bilayer nature of the coating improved hydrophobicity as the underlying CNT layer improved roughness and the FAS layer provided more hydrophobicity. The presence of CNT and FAS increased the water contact angle by 27.7%, 16%, and 17.5%, and the plasma contact angle by 29%, 21.7%, and 16.5% for glass, stainless steel, and PTFE surfaces, respectively. These findings pave the way for further research and development in the field of surface coatings, especially in biomedical applications, to improve material performance.

## Figures and Tables

**Table 1:** Water and plasma contact angles of FAS and FAS–CNT coatings.

Sample Types		Water Contact Angle (°)	Plasma Contact Angle (°)
Glass	Unmodified	20 ± 2°	20 ± 2°
	Coated with FAS	83 ± 1°	79 ± 1°
	Coated with FAS–CNT	106 ± 2°	102 ± 2°
Steel	Unmodified	80 ± 2°	67 ± 2°
	Coated with FAS	100 ± 3°	92 ± 3°
	Coated with FAS–CNT	116 ± 2°	112 ± 2°
PTFE	Unmodified	109 ± 2°	102 ± 2°
	Coated with FAS	120 ± 1°	115 ± 1°
	Coated with FAS–CNT	141 ± 2°	134 ± 2°

**Table 2:** Surface roughness parameters of the modified surfaces.

Sample Types		Roughness Parameters	
		Ra (nm)	Rq (nm)
Glass	Coated with FAS	0.804	2.18
	Coated with FAS–CNT	488	654.5
Steel	Coated with FAS	8.22	12.2
	Coated with FAS–CNT	301	394.5
PTFE	Coated with FAS	245.5	325
	Coated with FAS–CNT	284	359

## Data Availability

No new data were created or analyzed in this study.
